# Supplementary material for: Effectiveness of barber-facilitated “Doing What Matters in Times of Stress” intervention among urban literate youths in Western Kenya: A cluster randomised trial
Source: PLOS Glob Public Health. 2025 Jun 18;5(6):e0004712. doi: 10.1371/journal.pgph.0004712 (PMC12176197; doi:10.1371/journal.pgph.0004712)
Supplement: S1 Text — (DOCX) [file pgph.0004712.s001.docx]

## S1 Text. Tool for assessment of thoughts of suicide and impairment possibly due to severe mental, neurological or substance use disorder.

**ASSESSMENT OF THOUGHTS OF SUICIDE**

1. In the past month, have you had serious thoughts or a plan to end your life?

1. Yes
2. No

If yes, ask the participant to describe their thoughts or plans.

Write details here:

2. What actions have you taken to end your life?

3. Do you plan to end your life in the next two weeks?

1. Yes
2. No
3. Unsure

If yes or unsure, ask participant to describe their plan to you. Write details here:

**IMPAIRMENTS POSSIBLY DUE TO SEVERE MENTAL, NEUROLOGICAL OR SUBSTANCE USE DISORDERS**

The following items are based on your observations of the participant's behaviours. Do not ask the participant any questions here. Circle yes or no to indicate your observations and give details if needed.

1.Does the participant understand you (even though they speak the same language or dialect)?

(Can they understand basic words, questions or follow instructions?

1. Yes
2. No

If no, give details:

2. Is the participant able to follow what is happening in the assessment to a reasonable extent?

(E.g. can they recall recently discussed topics, do they understand who you are and what you are doing with them, do they understand to some extent why you are asking them questions? Please consider if the participant is so confused or drunk or high they cannot follow what is happening – then circle the response.)

1. Yes
2. No

If no, give details:

3. Are the participant's responses bizarre and/or highly unusual?

(E.g. uses made-up words, long periods of staring into space, talks to him/herself, stories are very bizarre or unbelievable.)

1. Yes
2. No

If yes, give details:

4. From the participant's responses and behaviours, does it appear that they are not in touch with reality or what is happening in the assessment?

(E.g. Delusions or firmly held beliefs or suspicions that do not make sense (they are bizarre) or are not realistic in the person's local context, or unrealistic paranoia, such as a highly unrealistic belief that someone is trying to harm them.)

1. Yes
2. No

If yes, give details

excluded participants whose response was NO on questions 1 or 2, or YES on questions 3 or 4.

Adopted from: [1]

**Reference**

1. WHO. Problem Management Plus (PM+): Individual psychological help for adults impaired by distress in communities exposed to adversity. Geneva, Switzerland 2018. Available: https://www.who.int/publications-detail-redirect/problem-management-plus-(-pm-)-individual-psychological-help-for-adults-impaired-by-distress-in-communities-exposed-to-adversity
