# Supplementary material for: Effectiveness of barber-facilitated “Doing What Matters in Times of Stress” intervention among urban literate youths in Western Kenya: A cluster randomised trial
Source: PLOS Glob Public Health. 2025 Jun 18;5(6):e0004712. doi: 10.1371/journal.pgph.0004712 (PMC12176197; doi:10.1371/journal.pgph.0004712)
Supplement: S2 Text — It provides detailed information on the study procedure. (DOCX) [file pgph.0004712.s002.docx]

# S2 Text. Intervention procedure

Barbers play a special role in men's and women's grooming. Beyond the grooming, there is an intangible element contributing significantly to their music experience. Its role in calming effect makes the grooming process smooth for both the barber and the client.

### Youth-friendly songs

We worked with 30 youths (other than the ones who participated in the study) and five local musicians from Western Kenya to develop mental health-themed music messaging that could resonate well with the youths. We then contracted five local musicians, two of whom were part of the content development, to produce mental health-themed music in local languages used in Western Kenya. The songs focused on mental health awareness, internalised mental health stigma, and experienced discrimination related to mental health. We also mapped existing local mental health-themed music and sought permission from the musicians to use the songs. The songs were delivered to the recruited and trained barbers of the project to play them in their barbershops. As the youth were having their hair cuts or visiting the barbershop to relax or chat, they could listen to the music which played in the background. The selected music played a pivotal role in setting the tone of the entire establishment. The music aimed to sensitise the youth and create an open discussion between the barber and the youth client on mental health, create awareness of mental health, and address the stigma associated with mental health. It also aimed to act as the barbers' entry point for introducing DWM to the youths.

### Recruitment of barbershops

The Project Coordinator and two Research Assistants visited barbershops within Bungoma and Kitale towns, talking to the barbershop owners and the barbers regarding the project. We also asked the barbers to share the information with colleagues, who were not reached physically by the research team, through their WhatsApp group or networks. Those interested could contact the Project Coordinator for details and recruitment. We recorded the contact details of barbers interested in the project and invited them for training.

**Training**

We hosted a five-day training in each of the two towns. The training involved in-class and field training led by a licensed psychologist supervised by a senior Mental Health and Psychosocial Support (MHPSS) specialist, who is a trainer of trainers (TOT) in DWM and clinical supervision while supported by the Project Lead and the Project Coordinator. The barbers were trained on basic helping skills, the rationale for each of the five intervention strategies and the session implementation of three face-to-face DWM sessions. To ensure required skills acquisition role-plays and regular supervision during DWM implementation were applied. We also trained them on self-care, common mental health conditions, and research ethics. The training content was adapted from the DWM guided manual by WHO [1].

During day one of the training, we introduced the barbers to mental health. We took them through the common mental health disorders among youths, confidentiality in intervention delivery and basic helping and self-care skills. Vignettes and case scenario questions were used to assess participants' understanding. On the second day, we introduced the barbers to the DWM protocol, participants' eligibility criteria, the qualities required of them as helpers, and their roles. During the last three days, we trained the barbers on the five intervention sections (grounding, unhooking, acting on your values, being kind and making room). We used the WHO DWM manual in the training [2]. We focused on the activities for each section, with barbers taking turns to role-play the different components of the intervention and assuming the three different meeting sessions with the youth clients. Role-play sessions took two hours long daily, followed by a 30-minute group discussion. During these four days, we also focused on self-care. The training enhanced the barbers' self-care techniques, and each barber identified a self-care plan, which was reviewed during individual or group supervision. Clinical supervision was also considered a process of building confidence and providing them with a safe and supportive environment to critically reflect on their attitudes towards people in mental distress.

During the last training day, we took them through crucial elements of the study procedure, including referral pathways for those with severe mental health conditions and how they would work with the data collectors (Research Assistants) in the intervention delivery. We repeatedly used role play to gauge and enhance understanding.

The barbers delivered the intervention with daily supervision by the project psychologist for the first week to ensure they did it correctly. After that, we had weekly support supervision by the project psychologist. Supervision ensured the quality delivery of the intended intervention and prevented helpers’ burnout and further, assisted barbers in feeling supported and confident with dealing with the clients.

## References

1. WHO. Doing What Matters in Times of Stress. Guided Self Help Manual 2.0. Field Test Version. Geneva, Switzerland 2020.

2. WHO. Doing What Matters in Times of Stress: An Illustrated Guide. Geneva, Switzerland: World Health Organization, Department of Mental Health and Substance Use; 2020. Available from: <https://www.who.int/publications/i/item/9789240003927>.
