# Supplementary material for: Effectiveness of barber-facilitated “Doing What Matters in Times of Stress” intervention among urban literate youths in Western Kenya: A cluster randomised trial
Source: PLOS Glob Public Health. 2025 Jun 18;5(6):e0004712. doi: 10.1371/journal.pgph.0004712 (PMC12176197; doi:10.1371/journal.pgph.0004712)
Supplement: S3 Text — Provides information on all outcome measures used in the study, their validation and scoring. (DOCX) [file pgph.0004712.s003.docx]

# S3 Text. Outcomes measures and their scoring

**Depression**

We used the Patient Health Questionnaire (PHQ-9) tool to assess for Depressive symptoms in the previous two weeks. The PHQ-9 is a nine-item self-reported scale scored on a 4-point Likert scale with scores ranging from 0 to 3 for each item. The total score of the PHQ-9 ranges from 0 to 27 [1,2]. English Version PHQ-9 has been validated among Kenyan adolescents, showing high internal consistency (Cronbach's α= 0.86), sensitivity of 95.0% and specificity of 73.0% [3]. Besides, among the adult population in Western Kenya, PHQ-9 was demonstrated to have culturally relevant content validity, high internal consistency (Cronbach alpha = 0.78), high sensitivity (85%) and specificity (95%) for depressive disorder diagnosis [4].

We scored PHQ-9 by adding scores for each question, with each question having a maximum score of 3 and a minimum of 0. The maximum score on PHQ-9 was 27, while the minimum was 0.

**Anxiety**

We measured probable anxiety in the previous two weeks using the Generalised Anxiety Disorder-7 (GAD-7) Scale. It has seven items scored on a four-point Likert scale ranging from zero (not at all) to three (nearly every day) [5]. It had good internal consistency α = 0.82 and acceptable test-retest reliability (ICC = 0.70) among adults living with HIV in Kilifi, Kenya [6]. The English version also had high internal consistency and validity among nurses and community health volunteers in Kenya [7].

We determined the individual GAD-7 score by adding scores for each question, ranging from 0 to 3 per question and a maximum total score of 21.

**Stress**

The Perceived Stress Scale (PSS-10) was used. Each item has a scale range of zero to four [8]. While we did not find information regarding its validation in Kenya, it has been widely used in the Kenyan context [9,10]. It also showed high content validity and reliability in a study among university students in the related context of Ethiopia [11].

For PSS-10, we first reverse-coded four positively stated items (items 4, 5, 7 and 8) (i.e. 0 => 4; 1 => 3; 2 => 2; 3 => 1; 4 => 0). We then determined the total individual score by summing the total score for each question per individual. Each question had a minimum score of 0 and a maximum of 4, hence a total maximum score of 40.

**Personally-identified problems**

We measured personally-identified problems using the Psychological Outcome Profile (PSYCHLOPS), a sensitive measure of change in participants' identified problems after intervention with high internal reliability [12,13]. It assesses the intervention effect on problems not evaluated by other standard outcome measures, hence being valuable for contexts like Kenya, where people have multiple problems [14]. It was used in the Kenyan context with an internal consistency of 0.64 [14,15].

PSYCHLOPS scores were calculated by summing the scores for questions 1 to 4, each with a six-point scale ranging from zero to five. Hence, the total maximum score for PSYCHLOPS was 20. If both Q1b (Problem 1) and Q2b (problem 2) were completed, the sum was determined by adding the scores for all four questions (Q1b + Q2b + Q3b + Q4). However, where one of the problem questions (problem 1) was completed and the other (Problem 2) was omitted, the total score was determined by doubling the score for problem 1 (Q1b) and adding the scores for questions three and four; (Q1b x 2) + Q3b + Q4.

**Functioning**

We used the 12-item interviewer-administered version of the WHO Disability Assessment Schedule 2.0 (WHODAS 2.0) to assess functional impairment covering mobility, cognition, socialising, self-care, and life activities within 30 days before the assessment. Its scores range from 0 to 48, with a high score indicating greater impairment severity. It has high sensitivity, strong criterion and construct validity [16]. It was previously used in Kenya [14,17] with an internal consistency of 0.66 [14].

For WHODAS 2.0, we calculated total individual scores by summing the scores for the twelve questions numbered S1 to S12. Scores for questions H1, H2 and H3 were recorded separately.

## **Resilience**

We measured resilience using the Brief resilience scale (BRS). It is a self-report questionnaire designed to measure an individual's ability to bounce back or recover from stress and adversity. It consists of six items that assess how individuals typically respond to stressors or setbacks in their lives. Respondents rate each item on a 5-point Likert scale, ranging from 1 (strongly disagree) to 5 (strongly agree) [18]. BRS has high structural and internal reliability and construct validity [19].

We reverse-coded the scores for items 2, 4, and 6 on the BRS scale. We then determined the total score by summing the individual questions' scores. With each of the six items on the scale having a score ranging from 1 to 5, the total maximum score on the scale was 30. We then divided the total individual score by the total number of questions completed by the participant to get the average individual score for the BRS scale.

**References**

1. Kroenke K, Spitzer RL, Williams JB. The PHQ-9: validity of a brief depression severity measure. J Gen Intern Med. 2001;16(9):606-13. https://doi.org/10.1046/j.1525-1497.2001.016009606.x

2. Kroenke K, Spitzer RL, Williams JB, Löwe B. The Patient Health Questionnaire Somatic, Anxiety, and Depressive Symptom Scales: a systematic review. Gen Hosp Psychiatry. 2010;32(4):345-59. https://doi.org/10.1016/j.genhosppsych.2010.03.006

3. Tele AK, Carvajal-Velez L, Nyongesa V, Ahs JW, Mwaniga S, Kathono J, et al. Validation of the English and Swahili Adaptation of the Patient Health Questionnaire-9 for Use Among Adolescents in Kenya. J Adolesc Health. 2023;72(1s):S61-s70. https://doi.org/10.1016/j.jadohealth.2022.10.003

4. Monahan PO, Shacham E, Reece M, Kroenke K, Ong'or WO, Omollo O, et al. Validity/reliability of PHQ-9 and PHQ-2 depression scales among adults living with HIV/AIDS in western Kenya. J Gen Intern Med. 2009;24(2):189-97. https://doi.org/10.1007/s11606-008-0846-z

5. Spitzer RL, Kroenke K, Williams JB, Löwe B. A brief measure for assessing generalized anxiety disorder: the GAD-7. Arch Intern Med. 2006;166(10):1092-7. https://doi:10.1001/archinte.166.10.1092

6. Nyongesa MK, Mwangi P, Koot HM, Cuijpers P, Newton C, Abubakar A. The reliability, validity and factorial structure of the Swahili version of the 7-item generalized anxiety disorder scale (GAD-7) among adults living with HIV from Kilifi, Kenya. Ann Gen Psychiatry. 2020;19:62. https://doi.org/10.1186/s12991-020-00312-4

7. Odero SA, Mwangi P, Odhiambo R, Nzioka BM, Shumba C, Ndirangu-Mugo E, Abubakar A. Psychometric evaluation of PHQ-9 and GAD-7 among community health volunteers and nurses/midwives in Kenya following a nation-wide telephonic survey. Front Psychiatry. 2023;14:1123839. https://doi: 10.3389/fpsyt.2023.1123839

8. Cohen S. Perceived stress in a probability sample of the United States. In S. Spacapan & S. Oskamp (Eds.), The social psychology of health (pp. 31–67). Sage Publications, Inc. 1988.

9. Samia P, Premji S, Tavangar F, Yim IS, Wanyonyi S, Merali M, et al. Adverse Childhood Experiences and Changing Levels of Psychosocial Distress Scores Across Pregnancy in Kenyan Women. Int J Environ Res Public Health. 2020;17(10). https://doi.org/10.3390/ijerph17103401

10. Shah S, Laving A, Okech-Helu VC, Kumar M. Depression and its associated factors: perceived stress, social support, substance use and related sociodemographic risk factors in medical school residents in Nairobi, Kenya. BMC Psychiatry. 2021;21(1):444. https://doi.org/10.1186/s12888-021-03439-0

11. Tsegaye BS, Andegiorgish AK, Amhare AF, Hailu HB. Construct validity and reliability Amharic version of perceived stress scale (PSS-10) among Defense University students. BMC Psychiatry. 2022;22(1):691. https://doi.org/10.1186/s12888-022-04345-9

12. Ashworth M, Evans C, Clement S. Measuring psychological outcomes after cognitive behaviour therapy in primary care: a comparison between a new patient-generated measure “PSYCHLOPS”(Psychological Outcome Profiles) and “HADS”(Hospital Anxiety and Depression Scale). Journal of Mental Health. 2009;18(2):169-77. https://doi.org/10.1080/09638230701879144

13. Ashworth M, Robinson SI, Godfrey E, Shepherd M, Evans C, Seed P, et al. Measuring mental health outcomes in primary care: the psychometric properties of a new patient-generated outcome measure,'PSYCHLOPS'('psychological outcome profiles'). Primary care mental health. 2005;3(4). ISSN: 14764717

14. Bryant RA, Schafer A, Dawson KS, Anjuri D, Mulili C, Ndogoni L, et al. Effectiveness of a brief behavioural intervention on psychological distress among women with a history of gender-based violence in urban Kenya: A randomised clinical trial. PLoS Med. 2017;14(8):e1002371. https://doi.org/10.1371/journal.pmed.1002371

15. Harper Shehadeh M, Van't Hof E, Schafer A, van Ommeren M, Farooq S, Hamdani SU, et al. Using a person-generated mental health outcome measure in large clinical trials in Kenya and Pakistan: Self-perceived problem responses in diverse communities. Transcult Psychiatry. 2020;57(1):108-23. https://doi.org/10.1177/1363461519854831

16. Ustün TB, Chatterji S, Kostanjsek N, Rehm J, Kennedy C, Epping-Jordan J, et al. Developing the World Health Organization Disability Assessment Schedule 2.0. Bull World Health Organ. 2010;88(11):815-23. https://doi: 10.2471/BLT.09.067231

17. Chepngeno-Langat G, Madise N, Evandrou M, Falkingham J. Gender differentials on the health consequences of care-giving to people with AIDS-related illness among older informal carers in two slums in Nairobi, Kenya. AIDS Care. 2011;23(12):1586-94. https://doi.org/10.1080/09540121.2011.569698

18. Smith BW, Dalen J, Wiggins K, Tooley E, Christopher P, Bernard J. The brief resilience scale: assessing the ability to bounce back. Int J Behav Med. 2008;15(3):194-200. https://doi: 10.1080/10705500802222972.

19. Sánchez J, Estrada-Hernández N, Booth J, Pan D. Factor structure, internal reliability, and construct validity of the Brief Resilience Scale (BRS): A study on persons with serious mental illness living in the community. Psychol Psychother. 2021;94(3):620-45. https://doi.org/10.1111/papt.12336
