# Supplementary material for: Effectiveness of barber-facilitated “Doing What Matters in Times of Stress” intervention among urban literate youths in Western Kenya: A cluster randomised trial
Source: PLOS Glob Public Health. 2025 Jun 18;5(6):e0004712. doi: 10.1371/journal.pgph.0004712 (PMC12176197; doi:10.1371/journal.pgph.0004712)
Supplement: S1 Table — It presents the results from the sample size calculation indicating the required clusters and participants per cluster. (DOCX) [file pgph.0004712.s004.docx]

## S1 Table. Sample size calculation table

| Members Per Group | 9 | 10 | 11 | 12 | 13 |
| --- | --- | --- | --- | --- | --- |
| 8 | 0.7309 | 0.6877 | 0.6515 | 0.6205 | 0.5936 |
| 12 | 0.6078 | 0.5719 | 0.5418 | 0.5160 | 0.4937 |
| 15 | 0.5509 | 0.5184 | **0.4911** | 0.4677 | 0.4475 |
| 19 | 0.4980 | 0.4686 | 0.4439 | 0.4228 | 0.4045 |
| 23 | 0.4603 | 0.4331 | 0.4103 | 0.3908 | 0.3738 |
