# Supplementary material for: Effectiveness of barber-facilitated “Doing What Matters in Times of Stress” intervention among urban literate youths in Western Kenya: A cluster randomised trial
Source: PLOS Glob Public Health. 2025 Jun 18;5(6):e0004712. doi: 10.1371/journal.pgph.0004712 (PMC12176197; doi:10.1371/journal.pgph.0004712)
Supplement: S2 Table — This table presents Leave-One-Out Information Criterion (LOOIC) values for each Bayesian multilevel ordinal regression model. These values were used to evaluate best fit model for our data. (DOCX) [file pgph.0004712.s005.docx]

**S2 Table: Leave-One-Out Information Criterion (LOOIC) from the different Bayesian multilevel ordinal regression models.**

|  | **Depression** | | **Anxiety** |  | **Stress** |  | **Functioning** | | **Problem** |  | **Resilience** | |
| --- | --- | --- | --- | --- | --- | --- | --- | --- | --- | --- | --- | --- |
| **MODEL** | **LOOIC** | **SE** | **LOOIC** | **SE** | **LOOIC** | **SE** | **LOOIC** | **SE** | **LOOIC** | **SE** | **LOOIC** | **SE** |
| Model1: Bayesian cumulative ordinal model with Proportional odds assumption | 1043.00 | 38.42 | 1315.56 | 29.99 | 695.35 | 36.24 | 837.22 | 40.47 | 1534.51 | 38.02 | 747.26 | 34.61 |
| Model 2: Category-specific effects model using adjacent-category method | - | - | 1269.63 | 35.26 | 683.12 | 37.89 | 801.28 | 45.39 | - | - | 736.92 | 35.41 |
| Model 3: Unequal variances model with unequal variance in selected variables | 1026.47 | 37.42 | 1289.55 | 30.72 | 687.62 | 36.52 | 822.01 | 42.03 | 1531.81 | 37.81 | 750.51 | 35.21 |
| Model 3a: Unequal variances model with unequal variance in all covariates | 937.82 | 30.36 | 1289.11 | 32.83 | 696.26 | 38.24 | 834.47 | 42.95 | 1536.29 | 39.43 | 761.50 | 36.10 |
| Model 3b: Unequal variances model with unequal variance in all covariates +interaction term | 939.12 | 30.34 | 1288.98 | 32.83 | 697.25 | 38.48 | 834.57 | 43.29 | 1537.11 | 39.67 | 764.65 | 36.48 |
| Model 4: Adjacent-category model without category-specific effects | 1054.45 | 39.98 | 1312.32 | 29.65 | 696.73 | 36.53 | 842.89 | 41.77 | 1548.53 | 39.60 | 749.86 | 34.86 |
