# Supplementary material for: Effectiveness of barber-facilitated “Doing What Matters in Times of Stress” intervention among urban literate youths in Western Kenya: A cluster randomised trial
Source: PLOS Glob Public Health. 2025 Jun 18;5(6):e0004712. doi: 10.1371/journal.pgph.0004712 (PMC12176197; doi:10.1371/journal.pgph.0004712)
Supplement: S1 Checklist — (DOCX) [file pgph.0004712.s013.docx]

**S1 Checklist. CONSORT 2010 checklist for reporting of a cluster randomised trial**

| **Section/topic and item No** | **Standard checklist item** | **Extension for cluster designs** | **Page No*** |
| --- | --- | --- | --- |
| **Title and abstract** |  |  |  |
| 1a | Identification as a randomised trial in the title Identification as a cluster randomised trial in the title |  | 1 |
| 1b | Structured summary of trial design, methods, results, and conclusions. |  | 1 |
| **Introduction** |  |  |  |
| Background and objectives: |  |  |  |
| 2a | Scientific background and explanation of rationale | Rationale for using a cluster design | 5 |
| 2b | Specific objectives or hypotheses | Whether objectives pertain to the cluster level, the individual participant level, or both | 5 |
| **Methods** |  |  |  |
| **Trial design:** |  |  |  |
| 3a | Description of trial design (such as parallel, factorial) | Definition of cluster and description of how the design allocation ratio features apply to the clusters | 6 |
| 3b | Important changes to methods after trial commencement (such as eligibility criteria), with reasons |  | 8 |
| **Participants:** |  |  |  |
| 4a | Eligibility criteria for participants | Eligibility criteria for clusters | 7 |
| 4b | Settings and locations where the data were collected |  | 5-6 |
| **Interventions:** |  |  |  |
| 5 | The interventions for each group with sufficient details to allow the replication, | Whether interventions pertain to the cluster level, including how and when they were actually administered individual participant level, or both | 8-10 |
| **Outcomes:** |  |  |  |
| 6a | Completely defined prespecified primary and secondary outcome | Whether outcome measures pertain to the cluster measures, including how and when they were assessed level, the individual participant level, or both | 10 |
| 6b | Any changes to trial outcomes after the trial commenced, with reasons |  | NA |
| **Sample size:** |  |  |  |
| 7a | How sample size was determined | Method of calculation, number of clusters(s) (and  whether equal or unequal cluster sizes are assumed), cluster size, a coefficient of intracluster correlation (ICC or k), and an indication of its uncertainty | 6 |
| 7b | When applicable, explanation of any interim analyses and stopping guidelines |  | NA |
| **Randomisation** |  |  |  |
| **Sequence generation:** |  |  |  |
| 8a | Method used to generate the random allocation sequence |  | 8 |
| 8b | Type of randomisation; details of any restriction (such as blocking and block size) Details of stratification or matching if used |  | 8 |
| **Allocation concealment mechanism:** |  |  |  |
| 9 | Mechanism used to implement the random allocation sequence (such as sequentially numbered containers), describing any steps taken to conceal the sequence until interventions were assigned | Specification that allocation was based on clusters rather than individuals and whether allocation concealment (if any) was at the cluster level, the individual participant level, or both | 8 |
| **Implementation:** |  |  |  |
| 10 | Who generated the random allocation sequence, who enrolled participants, and who assigned participants to interventions | Replaced by 10a, 10b, and 10c | 8 |
| 10a |  | Who generated the random allocation sequence, who enrolled clusters, and who assigned clusters to interventions | 8 |

|  |  |  | **Page** |
| --- | --- | --- | --- |
| **Section/topic and item No** | **Standard checklist item** | **Extension for cluster designs** | **No*** |
| 10b |  | Mechanism by which individual participants were included in clusters for the purposes of the trial (such as complete enumeration, random sampling) | 8 |
| 10c |  | From whom consent was sought (representatives of the cluster, or individual cluster members, or both) and whether consent was sought before or after randomisation | 7 and 13 |
| **Blinding:** |  |  |  |
| 11a | If done, who was blinded after assignment to interventions (for example, participants, care providers, those assessing outcomes) and how |  | 9 |
| 11b | If relevant, description of the similarity of interventions |  | NA |
| **Statistical methods:** |  |  |  |
| 12a | Statistical methods used to compare groups for primary and secondary outcomes | How clustering was considered | 12 |
| 12b | Methods for additional analyses, such as subgroup analyses and adjusted analyses |  | 12 |
| **Results** |  |  |  |
| **Participant flow (a diagram is strongly recommended):** | |  | Fig 1 |
| 13a | For each group, losses and exclusions after randomisation, together with reasons | For each group, the numbers of clusters that were randomly assigned, received intended treatment, and were analysed for the primary outcome | 14 & Fig 1 |
| 13b | For each group, losses and exclusions after randomisation, together with reasons | For each group, losses and exclusions for both  clusters and individual cluster members | 14 & Fig 1 |
| **Recruitment:** |  |  |  |
| 14a | Dates defining the periods of recruitment and follow-up |  | 9 |
| 14b | Why the trial ended or was stopped |  | NA |
| **Baseline data:** |  |  |  |
| 15 | A table showing baseline demographic and clinical characteristics for each group | Baseline characteristics for the individual and cluster levels as applicable for each group | 16 |
| **Numbers analysed:** |  |  |  |
| 16 | For each group, number of participants (denominator) included in each analysis and whether the analysis was by original assigned groups | For each group, number of clusters included in each analysis | 16-17 |
| **Outcomes and estimation:** |  |  |  |
| 17a | For each primary and secondary outcome, results for each group, and the estimated effect size and its precision (such as 95% confidence interval) | Results at the individual or cluster level as applicable and a coefficient of intracluster correlation (ICC or *k*) for each primary outcome | 17-19 |
| 17b | For binary outcomes, presentation of both absolute and relative effect sizes is recommended |  |  |
| **Ancillary analyses:** |  |  |  |
| 18 | Results of any other analyses performed, including subgroup analyses and adjusted analyses, distinguishing prespecified from exploratory |  | 17-19 |
| **Harms:** |  |  |  |
| 19 | All-important harms or unintended effects in each group (for specific guidance see CONSORT for harms106) | | NA |
| **Discussion** |  | |  |
| **Limitations:** |  | |  |
| 20 | Trial limitations, addressing sources of potential bias, imprecision, and, if relevant, multiplicity of analyses | | 29 |
| **Generalisability:** |  | |  |
| 21 | Generalisability (external validity, applicability) of the trial findings | Generalisability to clusters and/or individua  participants (as relevant) | 29 |

|  |  |  | **Page** |
| --- | --- | --- | --- |
| **Section/topic and item No** | **Standard checklist item** | **Extension for cluster designs** | **No*** |
| **Interpretation:** |  |  |  |
| 22 | Interpretation consistent with results, balancing benefits and harms, and considering other relevant evidence |  | 29 |
| **Other information** |  |  |  |
| **Registration:** |  |  |  |
| 23 | Registration number and name of trial registry |  | 1 |
| **Protocol:** |  |  |  |
| 24 | Where the full trial protocol can be accessed, if available |  | S14_File |
| **Funding:** |  |  |  |
| 25 | Sources of funding and other support (such as supply of drugs), role of funders |  |  |
| *Page numbers optional depending on journal requirements. | |  |  |

**Adapted from**: Campbell et al. (2012).

Campbell MK, Piaggio G, Elbourne DR, Altman DG & CONSORT Group. Consort 2010 statement: extension to cluster randomised trials. Bmj. 2012 Sep 4;345. <https://doi.org/10.1136/bmj.e5661>
