# Supplementary material for: Effectiveness of barber-facilitated “Doing What Matters in Times of Stress” intervention among urban literate youths in Western Kenya: A cluster randomised trial
Source: PLOS Glob Public Health. 2025 Jun 18;5(6):e0004712. doi: 10.1371/journal.pgph.0004712 (PMC12176197; doi:10.1371/journal.pgph.0004712)
Supplement: S1 Protocol — (PDF) [file pgph.0004712.s014.pdf]

PUBLIC TITLE: WE MATTER TOO: A MENTAL HEALTH INTERVENTION FOR ADOLESCENTS AND YOUTHS USING AFRICAN HIP-HOP (BONGO) IN BARBER SHOPS IN KENYA

SCIENTIFIC TITLE: EFFECTIVENESS OF LOCAL HIP-HOP MUSIC AND DOING WHAT MATTERS IN TIMES OF STRESS IN ADDRESSING MENTAL HEALTH CHALLENGES AMONG THE YOUTHS IN WESTERN KENYA.

Sponsor: Sentum Scientific Solutions

Funding Agency: Grand Challenges Canada

ABSTRACT

Background: Globally, mental health (MH) challenges are increasing in prevalence, being common among youths and adolescents. This is also the case in Kenya, where available evidence has pointed to an increased prevalence of mental health disorders among young people. However, due to associated stigma and limited awareness of the MH conditions, affected individuals rarely seek mental health services. This is also partly due to the scarcity of such services and their providers. Hence, there is a need for culturally appropriate, community-based mental health services that are responsive to the needs of the youths to address the growing burden of mental health disorders among this group in Kenya.

Objectives: This is an implementation project that will seek to determine the effectiveness of mental health-themed music and Doing What Matters in times of stress (DWM) intervention in reducing mental health problems (psychological distress, anxiety, self-reported problems and depression) and improving the knowledge and attitude on mental health among youths in Western Kenya. Besides, the project will seek to determine the intervention's effectiveness on the resilience and functioning of the youths.

Methods: The project will be carried out in Bungoma and Kitale towns in Western Kenya, among youths (18-29 years) visiting selected barber shops for a routine haircut. The project will collaborate with local musicians to produce mental health-themed music to be played in selected barber shops. Selected barbers will be trained as facilitators in the delivery of doing what matters in times of stress (DWM). The barbers will use MH-themed music to initiate discussion on the project with youths visiting their barbershops. Those who consent will be screened for eligibility by research assistants, and eligible ones will be enrolled in the project by the barber. The barbers will help deliver the DWM intervention, which will last five weeks for each participant. Thereafter, selected participants will receive entrepreneurship and life skills training and support. A mixed-method approach involving a cluster randomized control trial design with each barber used as a cluster (40 clusters with 15 youths each); hence, a total of 600 youths (300 cases and 300 controls) and qualitative evaluation will be used for the evaluation. The 40 clusters will be randomised to either the intervention or control group using a randomisation ratio of 1:1. The project outcomes; depression, anxiety, stress, self-identified problems, resilience, functioning and mental health awareness will be assessed at baseline and post-intervention.

Background

Globally, one in seven adolescents experiences a mental disorder, accounting for 13% of the global burden of disease in this age group (WHO, 2021). African Youths are disproportionately affected by mental health disorders, with a median depression and anxiety prevalence of 26.9% and 29.8%, respectively, among youths in the general population (Jörns-Presentati et al., 2021).

The scenario is no different among Kenyan youths and adolescents, with a prevalence of 28.06% and 30.38% for depressive and anxiety symptoms, respectively, among school-going adolescents (Osborn et al., 2021). A high prevalence of moderate and severe depressive symptoms of 35.7% and 5.6%, respectively, has also been reported among university students in Kenya (Othieno et al. 2014).

While limited mental health services exist due to a shortage of professional mental health service providers in the country (Kwobah et al., 2023), a previous study found that youths rarely utilise mental health services, especially those provided at health facilities. A small proportion of those who seek such services do so when it is already late, with the mean time from the onset of symptoms to the time of accessing care at the clinic being 16.6 months (Kamau et al., 2017). Besides, most of the small proportion who sought such services were referred by medical professionals or teachers (Kamau et al., 2017).

Stigmatization and discrimination of people with mental health disorders are also rife in Kenya. Approximately 81.9% of those with mental disorders in Kenya had experienced discrimination in making or keeping friends, 69.7% in finding a job, 56.3% in keeping a job, and 63.3% in dating or having an intimate relationship (Ebuenyi et al., 2019). Anticipated discrimination stopped 59.2% from applying for work, 40.8% from applying for education or training courses, and 63.4% from having a close personal relationship. Females and unemployed individuals reported significantly higher experienced discrimination than males and employed individuals. The huge treatment gap, stigma and discrimination have been highlighted to worsen the burden of mental illness (Ebuenyi et al., 2019). Besides the existing cultural barriers, the punitive penal code, which criminalizes suicide and suicidal attempts, affects the prevention, reporting, and management of mental health cases (MOH, 2020).

Previous studies in Western Kenya reported the existence of gaps in mental health in the region (Kwobah et al., 2023; Olwanda, 2021). Inadequate staff, lack of information,

funding, infrastructure, and the unavailability of psychotropic medications were noted as barriers to the utilization of mental health services (Olwanda, 2021). This necessitates low-cost, culturally appropriate and lay-delivered interventions to address the prevalent mental health needs while at the same time surmounting the barriers of limited mental health providers, limited facilities, and resources for mental health services in the region.

Recognising the magnitude of the problem, there has been an increase in efforts to develop and test psychological therapies, especially community-based ones, aimed at addressing mental health disorders (McNeish et al., 2019), with Doing What Matters in times of stress (DWM), Self Help Plus and Problem Management Plus being examples of such interventions developed by WHO (WHO, 2020). However, most of such interventions have rarely been tested among youths.

Doing What Matters in Times of Stress (DWM; WHO, 2020) is a core part of the WHO's Self Help Plus (SH+) intervention (Epping-Jordan et al., 2016), a five-session stress management course that uses pre-recorded audio complemented with an illustrated self-help book adapted for the target cultural group and can be used for large groups of up to 30 people. DWM can be delivered as lay-guided self-help and is less costly and less time-consuming. The effectiveness of the more extensive SH+ course in reducing emotional distress and improving functionality was demonstrated in a large randomized controlled trial (RCT) among South Sudanese refugees in Uganda (Tol et al., 2020). It has also been shown to prevent mental health disorders among Syrian refugees in Turkey (Acarturk et al., 2022). To the best of our knowledge, the efficacy of DWM as a standalone intervention among youth has not been tested.

To help address this gap, Sentum Scientific Solutions will utilize barbershops as a site for creating mental health awareness and providing access to youth-friendly mental health services to youths in two counties of Kenya (Bungoma and Trans-nzoia). It is projected that the intervention will help address the mental health crisis among youths in Western Kenya, which has received less attention. This project will utilise the Doing What Matters in Times of Stress (DWM) intervention to target youths experiencing mild or moderate psychological distress.

Aims and objectives

Broadly, the project will seek to determine the feasibility and effectiveness of using local music and barber facilitated DWM to improve mental health outcomes and well-being in Western Kenya.

Specifically, the project will aim to test 1) the feasibility of using hip-hop and rap music to initiate a conversation on mental health among youths, 2) the effectiveness of the barber-delivered DWM intervention in improving mental health outcomes (depression, anxiety, stress, self-identified problems, functioning, and resilience) among participants 3) changes in knowledge, and attitude towards mental health among the youths involved in the intervention and 4) the impact of the entrepreneurship and life skills training on economic outcomes and wellbeing of the participants.

Methods and materials

Study design

A mixed-method study approach will be used with both quantitative and qualitative study designs. For the quantitative part, the project will utilise a two-arm, cluster randomised control trial design with an intervention and waiting list control group, with two outcomes assessment points: preintervention and post-intervention, which will be done after the five-week intervention. The qualitative design will mainly be used for process evaluation to gather in-depth information regarding the role of music in the project, understand participants' knowledge and attitude towards mental health, and assess the participants' engagement and satisfaction with the project.

Project setting

We will implement the project in Kitale, Trans-Nzoia County and Bungoma Town, Bungoma County, within Western Kenya. We selected these sites because of limited mental health services and infrastructure (Olwanda, 2021).

We will purposely focus the project on the major towns of the two counties (Bungoma and Kitale town), where most youths are concentrated. This project will consist of four components: creating mental health awareness, identifying youth with mild and moderate psychological distress, delivering a low-cost DWM intervention with barbers as helpers/facilitators and socio-economic empowerment of selected youths with mild or moderate mental health distress who identified economic difficulties or lack of employment as their main problem.

We will implement the project in barbershops in the two study towns. Barbershops have been identified as potential areas where men securely share their views on various topics ranging from personal issues to the economy to politics, and they serve as bonding areas (Mbilishaka, 2018).

Target population

The study will target youths aged 18 to 29 years living in urban areas of Kitale town of Trans-Nzoia county and Bungoma town in Bungoma county. The age group of 18-29 years was selected based on the scarcity of data regarding interventions targeting this age group in the targeted settings.

A total of 40 barbershops and barbers will be included in the study (20 barbershops per county). The barbershops will be invited to participate. The barbers/barbershops will act as the study clusters.

Sample size

The target sample size for this study will be 40 barbers/barbershops (clusters) with 15 youths from each, translating to 600 youths (300 youths in the intervention group and 300 youths in the control group). The cluster-randomised trial sample calculator based on the National Institutes of Health will be used (https://researchmethodsresources.nih.gov/). The sample size will be determined with the ability to detect as low as Cohens d of 0.40, while taking into account an attrition rate of 20%. A statistical power of 0.8 and two-tailed alpha of 0.05 with a correlation coefficient of 0.5 between baseline and endpoint scores will be assumed. An interclass correlation of 0.02 will be assumed to allow for possible clustering effects.

Eligibility criteria

Youths Inclusion criteria

1. Youths aged 18-29 years seeking for services from the selected barbershops.
2. Those who consent to participate in the project.
3. Those with non-severe psychological distress (PHQ-9 Score ≥ 5 to <15) and with functioning impairment (WHODAS score ≥17).

Exclusion criteria

1. Those with plans to end their lives within two weeks of the pre-assessment, hence imminent suicide attempt.
2. Those judged to have a neurological or severe mental disorder, substance use disorder or cognitive impairment.
3. Those who plan to relocate from the area within the project period.

Participants will be assessed for severe mental health disorders and neurological or substance use disorders based on a four-scale observation yes/no screening tool. A three-scale self-reporting suicide assessment scale will be administered to screen for suicidal thoughts. (Appendix III). This severe mental health neurological disorder screening tool is commonly used in community mental health intervention settings (Tol et al., 2020; Musotsi et al., 2022) and was used in the Kenyan population in a Problem Management Plus (PM+) project (Bryant et al., 2017).

The possible presence of severe mental disorders and/or cognitive impairment screening tool will not be verbally administered but reported based on observations of the research assistant undertaking screening assessments.

Those with suicide plans or severe mental health disorders will receive Psychological First Aid from the project clinical psychologist or Psychologist Research Assistants, plus referral for clinical diagnosis and management at existing public mental health facilities.

Barbers' eligibility criteria

We will select barbers to help the project and serve as facilitators to deliver the DWM intervention. Most are young people, with whom the visiting youths have already developed an existing rapport, hence ease of interaction.

The project team will visit the barbershops within the two study towns, discussing the project with the barbers and barbershop owners. With the barbershop owners' consent, we will recruit barbers aged 18-30 years who can read and write in English and are willing to participate in the project.

We will recruit one barber per shop so as not to interfere with normal barbershop functioning.

Participant recruitment

Youths will be approached by the trained barbers while visiting the barber shop for their routine haircut. The overall recruitment approach is to use music as a strategy to open dialogue on mental health between the trained barber and the youth before recruitment.

Intervention

Our intervention will have four components centred around the objectives. First, we will develop and use local mental health-themed music to create awareness and destigmatize mental health. The second component will involve the screening for functioning using World Health Organization Disability Assessment Schedule (WHODAS) (WHO, 2010;Ustün et al., 2010), symptoms of mental health conditions using psychometric measures Patient Health Questionnaire-9 (PHQ-9) (Kroenke et al., 2001; Kroenke et al., 2010), Psychological Outcome Profiles (PSYCHLOPS) (Ashworth et al., 2009; Ashworth et al., 2005), and Generalized Anxiety Disorder-7 (GAD-7) (Spitzer et al., 2006), Perceived Stress Scale 10 (PSS-10) (Cohen & Williamson, 1988), and resilience using Brief Resilience scale (BRS) (Smith et al., 2008). The third component will involve the use of music to initiate conversation between the barber and the youth on mental

health and the delivery of DWM interventions at the barbershop, considered a youth-friendly space, where trained and supervised barbers will facilitate the delivery of DWM to youths with mild or moderate psychological distress. The last component will involve an entrepreneurship intervention targeting selected youths with mild or moderate mental health included in the second component.

Music development

MHPSS experts, youths, and local musicians will collaborate to develop mental health-themed music messaging that is appropriate for the targeted youths and provides essential mental health awareness. The contracted musicians will then use the messages to compose the songs. The music will be pre-tested and validated with selected youth and refined based on feedback.

After pre-testing and validation, four local hip-hop musicians will be engaged and facilitated to produce four mental health-themed music (1 per musician) in local languages. This music will be routinely played in select barber shops in 2 counties of Kenya where the project will be conducted.

The focus of these songs will be on mental health awareness, internalized stigma and experienced discrimination. The songs will be used by the barber to create an open discussion with the youth client and awareness of mental health and mental health stigma reduction. The barbers will use these songs to initiate a conversation about the project with eligible youths visiting their shops. Those willing to participate will be recruited by the barber and referred to the study research assistant, who will carry out screening and project pre-assessment. Those found eligible will be recruited and included in the project after consenting. Those found to have severe mental health conditions will be referred to public health facilities or related projects offering services for severe mental health conditions for diagnosis and management.

Randomization

Cluster randomization will be used with the cluster defined as barbers/barbershops, and all participants recruited and offered intervention by each barber randomized to the same group as he barber (either intervention or control group). Randomization at the level of individual youth will not be feasible owing to the danger of contamination.

Allocation

A total of 40 clusters, each with 15 participants, is targeted. A total of 20 clusters will be randomly allocated to the DWM intervention group, while the other 20 clusters, with 15 members each, will be randomly assigned to the waiting list control (WLC) group. A simple randomisation approach will be used, either using randomization software or simple manual approaches.

Blinding

Single blinding will be applied with only the data collectors/outcome assessors and the biostatistician analysing the data blinded of the participant's/barbers group allocation. Due to the nature of the intervention, it is not possible to blind participants and the barbers delivering the intervention.

Intervention procedure

We will train the barbers on basic mental health information, helping skills and regarding the DWM intervention. We will use the trained barbers as helpers to deliver the DWM intervention. Doing What Matters in Times of Stress intervention booklets and audio developed by WHO (WHO, 2020) will be used by the trained barbers as intervention material.

The trained barbers will meet the participants in three meetings:

Introductory Appointment: this will cover an introduction to the stress management guide, a discussion of interest, assessment, and planning. This session will take up to one hour.

One-week follow-up: This will cover using the stress management guide and support with overcoming barriers or problems with use and clarifying concepts. This will take upto 50 minutes.

One-month follow-up: This last session will cover the use of the DWM stress management guide, support overcoming barriers or problems with use, planning for continued future use, and completing follow-up assessment and goodbye or referral. This will also take approximately one hour. After the meetings, the youths in the intervention will be expected to engage with the provided intervention audio and illustration guides on their own. In case of any difficulties, they will be free to contact and engage the trained barbers to explanation and help in understanding the content. The intervention supported by the barber will last for five weeks for each participant, after which the Research

Assistants will conduct a post-intervention assessment. The intervention will be delivered in English.

The project will run a small validation exercise to verify understanding and develop a catalogue for words used in the local vernacular language that may not be well understood if spoken in English. The barbers will be trained always to ensure the participants understand the content. A word catalogue will be written to help each barber use the correct idioms based on the vernacular of the participants.

Entrepreneurship and life skills intervention component

A small sample of the youths (100) will be included in this component. These participants will be randomly selected from participants in the DWM intervention (50) and waiting list control (50) group participants. Selection will be based on having reported economic hardship or unemployment as the main problem faced during pre-assessment with the PSYCHLOPS tool. The selected youth will be invited for two-day entrepreneurship and life skills training.

After training, the youths will be asked to come up with business ideas in the area of interest they would like to pursue related to their area of skills. They will then be paired in groups based on similar interests and given interest-free micro-loans to start small businesses. They will also receive personalised mentorship from experienced entrepreneurship and apprenticeship experts. Those offered micro-loans will be required to keep records of their expenditure and income. They will be required to repay the loan in equal monthly instalments from the third month after the loan has been disbursed to them. Research assistant or project coordinator will follow up monthly with them and gather data on the performance of their businesses and income flow based on their records. The repaid amount will be loaned to other youths in a revolving manner.

As part of the intervention evaluation, in-depth qualitative interviews will be conducted to understand their views on the entrepreneurship component, including its usefulness, shortcomings, and aspects they would like improved.

Control group

A waiting list control group will be used. They will be assessed at baseline and endline (5 weeks from the date of baseline assessment). They will receive the intervention after the intervention is complete in the intervention group.

Study outcomes and outcomes measures

The effectiveness of DWM in reducing psychological distress is the primary aim and will involve the following outcome measures.

ii) **Stress, anxiety, depression and personally identified problems**

We will use Generalized Anxiety Disorder 7-item (GAD-7) for anxiety assessment, Patient Health Questionnaire-9 (PHQ-9) for depression and Perceived Stress Scale (PSS-9) for stress. In a study among adolescents and young people (AYP) in Nairobi and Kiambu counties in Kenya, both the PHQ-8 ($\alpha = 0.78$) and GAD-7 ($\alpha = 0.82$) showed good internal consistency and goodness of fit (Osborn et al., 2021).

The GAD-7 has been used and has met psychometric standards in prior studies with Kenyan adolescents (Osborn et al., 2020; Osborn et al., 2021). Cronbach α was 0.82 in a Kenyan adolescent sample (Osborn et al., 2020).

We will assess personally identified problems using the Psychological Outcome Profile (PSYCHLOPS), a sensitive measure of change in participants' identified problems after intervention with high internal reliability (Ashworth et al., 2009).

The secondary outcome will be the effectiveness of the intervention in improving functioning and resilience and will involve the following outcome measures.

i) **Functioning**

The functioning of the participants will be assessed using the WHO Disability Assessment Schedule (WHODAS 2.0) tool, developed by WHO to assess health and disability. It has six domains: mobility, self-care, life activities, getting along, cognition and participation. The 12-item version will be used in this study (WHO, 2016). The WHODAS 2.0 measures activity function and participation in daily activities in the 30 days preceding its application (WHO, 2010; Üstün et al., 2010). The assessment will be done pre- and post-intervention.

ii)**Resilience -measured using the Brief Resilience Scale**

The participants' level of resilience will be assessed using the Brief Resilience Scale (Smith et al., 2008).

Data collection process

Quantitative data collection

The data will be collected by trained research assistants who administer the questionnaire face-to-face. Each interview will last approximately 30 minutes. KoboTool box will be used for data collection using tablets, and no physical papers will be used during the process.

A questionnaire assessing participants' demographic information, including age, gender, area of residence, the highest level of education, and work status, will be administered at pre-test followed by the outcome measure questionnaires: PHQ-9 for depression, GAD-7 for anxiety, PSS-10 for stress, PSYCHLOPS for self-identified problems, BRS for resilience, and WHODAS for functioning and knowledge and attitude on mental health questionnaire.

Data analysis

The collected quantitative data in the Kobotool box will be imported into the Statistical Package for Social Sciences (SPSS) Version 27.0, where it will be coded and ready for analysis. Both per protocol and Intention to treat analysis (ITT) will be used in the analysis.

For descriptive analysis, mean and standard deviation or median and corresponding interquartile range will be used for continuous variables such as age, scores on depression, anxiety, stress, WHODAS, and BRS scales. For categorical variables such as gender and level of education, frequencies and percentages will be used to describe them.

To compare baseline characteristics between the intervention and control groups, $\chi 2$ tests for comparison of frequencies and independent-sample t-tests for comparison of mean scores on normally distributed continuous measures or the Mann-Whitney U-test for comparison of the median for skewed continuous variables will be used.

The possible impact of missing data will be examined via sensitivity analyses of augmented data sets, and missing data will be handled through modern imputation techniques such as multiple imputation.

A mixed-effect linear model will be used to determine the effectiveness of the intervention, controlling for participants' sociodemographic characteristics found to be significantly different between the two groups, and the pre-assessment outcome scores.

The significance level based on a two-sided will be taken as $P < 0.05$ at 95% confidence intervals.

For mental health outcome measures, we will also use the Reliable Change Index (RCI) and Clinically Significant Change (CSC) approaches, classifying participants as 'recovered' or 'improved', 'unchanged' and 'deteriorated' for each outcome (Jacobson & Truax, 1991). These approaches comprise measures of whether the change in scores is larger than expected due to outcome measure reliability and whether participants shift from clinical to non-clinical.

Qualitative data collection and analysis

A purposive sample of 12 participants will be used (8 youths involved in the intervention and four barbers engaged in its delivery). However, the sample will be increased or reduced based on the attainment of conceptual saturation when no new information is forthcoming.

In-depth interviews will be conducted with barbers and project participants (youths). Two different pre-prepared interview guides for youths and barbers will be used during the interview process. The guides contain open-ended questions. For barbers, the interview will seek to determine their experience delivering the intervention, the challenges encountered, and how it can be improved in the future. The interview guide for the youths will contain open-ended questions on their views on mental health-themed music, any role it played in their participation in the project, what they learned from the music, their experiences and engagement with the intervention, satisfaction, challenges faced, and any improvements needed.

A trained research assistant, competent in in-depth interviewing and qualitative research, will conduct the interviews. The interviews will be conducted either in Swahili or English based on the participants' preferences and capabilities. The interviews will be tape-recorded with the participant's consent.

The qualitative data will be transcribed verbatim before analysis using thematic analysis, which involves identification, analysis, and reporting patterns within data, resulting in the minimal organisation and minimal description of the data in rich detail (Braun and Clarke, 2006), will be used. In the process, an inductive approach will be taken, where the analysis

is data-driven without trying to fit it into any pre-existing framework (Thomas, 2006). The thematic analysis process will be based on Braun and Clarke (2006).

Ethical considerations

The protocol for this study will be sent to Moi University/ Moi Teaching and Referral Hospital (MTRH) Institutional Research Ethics Committee (IREC) for ethical review and approval. Permit to conduct the study in the selected regions will also be obtained from the National Commission for Science, Technology, and Innovation (NACOSTI) as local Kenyan laws require. The County Department of Health (CDOH) and the county commissioners of the study counties will be notified, and supporting letters will be obtained from their offices to help facilitate fieldwork.

Dissemination of findings

The study's findings will be shared with the county government of Bungoma and Trans-Nzoia health departments in the form of the final report and a short policy brief highlighting the key findings. The study results will be presented at local and international mental health conferences and published in reputable peer-reviewed journals.

Study timelines

	Dec 2022-Jan 2023	Feb-May 2023	May-June 2023	June 2023-April 2024	May-July 2024	Aug-Nov 2024
IRB Preparation/ Approval						
Fieldwork preparation and training						
DWM intervention delivery						
Economic empowerment component						
Report Preparation						

References

Acarturk, C., Kurt, G., Ilkkursun, Z., Uygun, E., & Karaoglan-Kahilogullari, A. (2022). "Doing What Matters in Times of Stress" to Decrease Psychological Distress During COVID-19: A Randomised Controlled Pilot Trial. *Intervention Journal of Mental Health and Psychosocial Support in Conflict Affected Areas*, *20*(2), 170-178.

Ashworth, M., Evans, C., & Clement, S. (2009). Measuring psychological outcomes after cognitive behaviour therapy in primary care: a comparison between a new patient-generated measure "PSYCHLOPS"(Psychological Outcome Profiles) and "HADS"(Hospital Anxiety and Depression Scale). *Journal of Mental Health*, *18*(2), 169-177.

Braun, V., & Clarke, V. (2006). Using thematic analysis in psychology. *Qualitative research in psychology*, *3*(2), 77-101.

Bryant, R. A., Schafer, A., Dawson, K. S., Anjuri, D., Mulili, C., Ndogoni, L., Koyiet, P., Sijbrandij, M., Ulate, J., Harper Shehadeh, M., Hadzi-Pavlovic, D., & van Ommeren, M. (2017). Effectiveness of a brief behavioural intervention on psychological distress among women with a history of gender-based violence in urban Kenya: A randomised clinical trial. *PLoS medicine*, *14*(8), e1002371. https://doi.org/10.1371/journal.pmed.1002371

Cohen, S., and G. Williamson. (1988). Perceived Stress in a Probability Sample of the United States. In *The Social Psychology of Health*, eds. S. Spacapan and S. Oskamp. Newbury Park, CA: Sage Publishers.

Ebuenyi, I. D., Regeer, B. J., Ndetei, D. M., Bunders-Aelen, J. F. G., & Guxens, M. (2019). Experienced and Anticipated Discrimination and Social Functioning in Persons With Mental Disabilities in Kenya: Implications for Employment. *Frontiers in psychiatry*, *10*, 181. https://doi.org/10.3389/fpsyt.2019.00181

Epping-Jordan, J. E., Harris, R., Brown, F. L., Carswell, K., Foley, C., García-Moreno, C., Kogan, C., & van Ommeren, M. (2016). Self-Help Plus (SH+): a new WHO stress management package. *World psychiatry : official journal of the World*

Psychiatric Association (WPA), *15*(3), 295–296. https://doi.org/10.1002/wps.20355

Jacobson, N. S., & Truax, P. (1991). Clinical significance: a statistical approach to defining meaningful change in psychotherapy research. *Journal of consulting and clinical psychology*, *59*(1), 12–19. https://doi.org/10.1037//0022-006x.59.1.12

Jörns-Presentati, A., Napp, A. K., Dessauvagie, A. S., Stein, D. J., Jonker, D., Breet, E., Charles, W., Swart, R. L., Lahti, M., Suliman, S., Jansen, R., van den Heuvel, L. L., Seedat, S., & Groen, G. (2021). The prevalence of mental health problems in sub-Saharan adolescents: A systematic review. *PloS one*, *16*(5), e0251689. https://doi.org/10.1371/journal.pone.0251689

Kamau, J. W., Omigbodun, O. O., Bella-Awusah, T., & Adedokun, B. (2017). Who seeks child and adolescent mental health care in Kenya? A descriptive clinic profile at a tertiary referral facility. *Child and adolescent psychiatry and mental health*, *11*, 14. https://doi.org/10.1186/s13034-017-0151-x

Kroenke, K., Spitzer, R. L., & Williams, J. B. (2001). The PHQ-9: validity of a brief depression severity measure. *Journal of general internal medicine*, *16*(9), 606–613. https://doi.org/10.1046/j.1525-1497.2001.016009606.x

Kroenke, K., Spitzer, R. L., Williams, J. B., & Löwe, B. (2010). The Patient Health Questionnaire Somatic, Anxiety, and Depressive Symptom Scales: a systematic review. *General hospital psychiatry*, *32*(4), 345–359. https://doi.org/10.1016/j.genhosppsych.2010.03.006

Kwobah, E. K., Turissini, M., Barasa, J., Kimaiyo, M., Okeyo, L., Araka, J., Njiriri, F., Matundura, R., & Jaguga, F. (2023). Mental healthcare services in Kenyan counties: a descriptive survey of four counties in Western Kenya. *BMC health services research*, *23*(1), 543. https://doi.org/10.1186/s12913-023-09481-w

Mbilishaka, Afiya Mangum. "Black Lives (and stories) Matter: Race narrative therapy in Black hair care spaces." *Community Psychology in Global Perspective* 4, no. 2 (2018): 22-33.

McNeish, R., Rigg, K. K., Tran, Q., & Hodges, S. (2019). Community-based behavioral health interventions: Developing strong community partnerships. *Evaluation and*

program planning, *73*, 111–115. https://doi.org/10.1016/j.evalprogplan.2018.12.005

Memiah, P., Wagner, F. A., Kimathi, R., Anyango, N. I., Kiogora, S., Waruinge, S., Kiruthi, F., Mwavua, S., Kithinji, C., Agache, J. O., Mangwana, W., Merci, N. M., Ayuma, L., Muhula, S., Opanga, Y., Nyambura, M., Ikahu, A., & Otiso, L. (2022). Voices from the Youth in Kenya Addressing Mental Health Gaps and Recommendations. *International journal of environmental research and public health*, *19*(9), 5366. https://doi.org/10.3390/ijerph19095366

MoH (2020). Kenya The Kenya Mental Health Policy 2015 Available online: https://publications.universalhealth2030.org/uploads/Kenya-Mental-Health-Policy. (accessed on December 2022)];

MoH (2022). Kenya Mental Health Taskforce Urges Government to Declare Mental Health a National Emergency Nairobi, Tuesday. 7 July 2020. Africa [(accessed on 8 December 2022)];2020 Available online: https://www.health.go.ke/mental-health-taskforce-urges-government-to-declare-mental-health-a-national-emergency-nairobi-tuesday-july-7-2020/

Musotsi, P., Koyiet, P., Khoshaba, N. B., Ali, A. H., Elias, F., Abdulmaleek, M. W., Simiyu, K & Rosenkranz, E. (2022). Highlighting complementary benefits of problem management plus (PM+) and doing what matters in times of stress (DWM) interventions delivered alongside broader community MHPSS programming in Zummar, Ninewa governorate of Iraq. *Intervention Journal of Mental Health and Psychosocial Support in Conflict Affected Areas*, *20*(2), 139-150.

NIH. Research Methods Resources: National Institutes of Health. [Accessed February 21st, 2023]. Available from: https://researchmethodsresources.nih.gov/.

Olwanda, E. (2021). Exploring barriers to the utilization of mental health services at public and private healthcare facilities in Vihiga sub-county, western Kenya: a case study of healthcare providers (Doctoral dissertation, Maseno university).

Osborn, T. L., Kleinman, A., & Weisz, J. R. (2021). Complementing standard western measures of depression with locally co-developed instruments: A cross-cultural

study on the experience of depression among the Luo in Kenya. *Transcultural psychiatry*, *58*(4), 499–515. https://doi.org/10.1177/13634615211000555

Osborn, T. L., Rodriguez, M., Wasil, A. R., Venturo-Conerly, K. E., Gan, J., Alemu, R. G., Roe, E., Arango G, S., Otieno, B. H., Wasanga, C. M., Shingleton, R., & Weisz, J. R. (2020). Single-session digital intervention for adolescent depression, anxiety, and well-being: Outcomes of a randomized controlled trial with Kenyan adolescents. *Journal of consulting and clinical psychology*, *88*(7), 657–668. https://doi.org/10.1037/ccp0000505

Osborn, T. L., Venturo-Conerly, K. E., Wasil, A. R., Schleider, J. L., & Weisz, J. R. (2020). Depression and anxiety symptoms, social support, and demographic factors among Kenyan high school students. *Journal of Child and Family Studies*, *29*(5), 1432-1443.

Othieno, C. J., Okoth, R. O., Peltzer, K., Pengpid, S., & Malla, L. O. (2014). Depression among university students in Kenya: prevalence and sociodemographic correlates. *Journal of affective disorders*, *165*, 120–125. https://doi.org/10.1016/j.jad.2014.04.070

Smith, B. W., Dalen, J., Wiggins, K., Tooley, E., Christopher, P., & Bernard, J. (2008). The brief resilience scale: Assessing the ability to bounce back. International Journal of Behavioral Medicine, 15(3), 194-200. http://dx.doi.org/10.1080/10705500802222972

Spitzer, R. L., Kroenke, K., Williams, J. B., & Löwe, B. (2006). A brief measure for assessing generalized anxiety disorder: the GAD-7. *Archives of internal medicine*, *166*(10), 1092–1097. https://doi.org/10.1001/archinte.166.10.1092

Thomas DR. (2006). A General Inductive Approach for Analysing Qualitative Evaluation Data. The American journal of evaluation.;27(2):237-46. https://doi.org/10.1177%2F1098214005283748

Tol, W. A., Leku, M. R., Lakin, D. P., Carswell, K., Augustinavicius, J., Adaku, A., Au, T. M., Brown, F. L., Bryant, R. A., Garcia-Moreno, C., Musci, R. J., Ventevogel, P., White, R. G., & van Ommeren, M. (2020). Guided self-help to reduce psychological distress in South Sudanese female refugees in Uganda: a cluster

randomised trial. *The Lancet. Global health*, *8*(2), e254–e263. https://doi.org/10.1016/S2214-109X(19)30504-2

Ustün, T. B., Chatterji, S., Kostanjsek, N., Rehm, J., Kennedy, C., Epping-Jordan, J., Saxena, S., von Korff, M., Pull, C., & WHO/NIH Joint Project (2010). Developing the World Health Organization Disability Assessment Schedule 2.0. *Bulletin of the World Health Organization*, *88*(11), 815–823. https://doi.org/10.2471/BLT.09.067231

WHO, (2010). World Health Organization. WHO Disability Assessment Schedule 2.0 (WHODAS 2.0). Measuring Health and Disability: Manual for WHO Disability Assessment Schedule (WHODAS 2.0). http://www.who.int/classifications/icf/whodasii/en/. /. https://www.who.int/standards/classifications/international-classification-of-functioning-disability-and-health/who-disability-assessment-schedule

WHO, (2021). Experts join forces for mental health in Kenya: World Health Organization. [cited 2024 20th April]. Available from: https://www.who.int/news-room/events/detail/2021/05/23/default-calendar/experts-join-forces-for-mental-health-in-kenya.

World Health Organization. (2016). Problem management plus (PM+): psychological help for adults in communities exposed to adversity: WHO Kenyan field-trial version 1.0, 2016 (No. WHO/MSD/MER/16.1). World Health Organization.

World Health Organization. (2020). Doing what matters in times of stress: an illustrated guide. World Health Organization, Department of Mental Health and Substance Use.

Wu, T., Jia, X., Shi, H., Niu, J., Yin, X., Xie, J., & Wang, X. (2021). Prevalence of mental health problems during the COVID-19 pandemic: A systematic review and meta-analysis. *Journal of Affective Disorders*, *281*, 91–98. https://doi.org/10.1016/j.jad.2020.11.117
