# Supplementary material for: Effectiveness of barber-facilitated “Doing What Matters in Times of Stress” intervention among urban literate youths in Western Kenya: A cluster randomised trial
Source: PLOS Glob Public Health. 2025 Jun 18;5(6):e0004712. doi: 10.1371/journal.pgph.0004712 (PMC12176197; doi:10.1371/journal.pgph.0004712)
Supplement: Attachment — Submitted filename: Response to editorial team comments_02_8_24.docx [file pgph.0004712.s015.docx]

**RESPONSE TO EDITORIAL TEAM COMMENTS**

Dear Editorial Team:

We are deeply thankful for your feedback. We have addressed the comment made on our manuscript as shown in the table below.

| **No** | **Comment** | **Response** | **Page** |
| --- | --- | --- | --- |
| **1** | Thank you for your response to our previous request for ethical approval extension documents. Although you correctly note that your approval period covers the recruitment of participants, we note that data collection continued until April 15th. Therefore, your ethics approval letter (Moi University) does not cover the entire date range for the study (31st July 2023 to 15th April 2024). Before we can proceed further with the submission, please provide the ethics approval extension document(s) for the study. If the document is in another language, please also provide an English translation. Please note that if this document is not included when your manuscript is resubmitted, it may be rejected. | We have attached the study approval extension letter granted by the ethics committee valid till March 2025. | **Abstract** |

Thank you for your valuable time in reading our manuscript.

We look forward to the feedback.

Yours sincerely,

Protus Yabunga

Corresponding Author
