# Supplementary material for: Effectiveness of barber-facilitated “Doing What Matters in Times of Stress” intervention among urban literate youths in Western Kenya: A cluster randomised trial
Source: PLOS Glob Public Health. 2025 Jun 18;5(6):e0004712. doi: 10.1371/journal.pgph.0004712 (PMC12176197; doi:10.1371/journal.pgph.0004712)
Supplement: Attachment — Submitted filename: Response to reviewers_12_10_24.docx [file pgph.0004712.s016.docx]

7/10/2024

Johanna Pruller, Ph.D.
Staff Editor
PLOS Global Public Health

Dear Dr Pruller,

I hope this message finds you well. I would like to express our gratitude for the opportunity to revise our manuscript titled *“Effectiveness of Barber-Facilitated "Doing What Matters in Times of Stress" Intervention Among Urban Literate Youths in Western Kenya: A cluster randomised trial”* (Manuscript ID: PGPH-D-24-01544) following the insightful comments from the reviewer.

We appreciate the time and effort the reviewer took to provide detailed feedback. Their comments were invaluable in strengthening the manuscript. Below, we have addressed each of the reviewers' points:

Journal Requirements:

1. **Comment:**

Your current Financial Disclosure does not have Grant Recipient. However, your funding information on the submission form indicates Grant Recipient “Protus Musotsi Yabunga". Please indicate by return email the full and correct funding information for your study and confirm the order in which funding contributions should appear. Please be sure to indicate whether the funders played any role in the study design, data collection and analysis, decision to publish, or preparation of the manuscript.

**Response:Here is the correct ifunding information**

This project was funded by Grand Challenges Canada Global Mental Health Program Grant (R-GMH-POC-2211-59472).

**The grant was awarded to**: Sentum Scientific Solutions

**Funding contribution:** Protus Musotsi Yabunga, Mary Nangukhula. (in correct order)

The funder of the study did not play any role in study design, data collection, data analysis, data interpretation or writing of the manuscript and in the decision to publish.

2**.Comment**

We note that your Data Availability Statement is currently as follows: "The data supporting the findings of this study will be made available open source. We are working on putting it on an open data repository."
 
 Please confirm at this time whether or not your submission contains all raw data required to replicate the results of your study. Authors must share the “minimal data set” for their submission. PLOS defines the minimal data set to consist of the data required to replicate all study findings reported in the article, as well as related metadata and methods (<https://journals.plos.org/plosone/s/data-availability#loc-minimal-data-set-definition>).
 
 For example, authors should submit the following data:
 
 - The values behind the means, standard deviations and other measures reported;
 - The values used to build graphs;
 - The points extracted from images for analysis.
 
 Authors do not need to submit their entire data set if only a portion of the data was used in the reported study.
 
 If your submission does not contain these data, please either upload them as Supporting Information files or deposit them to a stable, public repository and provide us with the relevant URLs, DOIs, or accession numbers. For a list of recommended repositories, please see <https://journals.plos.org/plosone/s/recommended-repositories>.

**Response:**

We have added the supporting dataset as an attachment to this submission as a Supporting Information file. **S2 file**

 

3**. Comment**

Please provide separate figure files in .tif or .eps format.

For more information about figure files please see our guidelines:

<https://journals.plos.org/globalpublichealth/s/figures> 
<https://journals.plos.org/globalpublichealth/s/figures#loc-file-requirements>

**Response:**

We have provided the supporting figures as .tif files formatted using pace as recommended.

Additional Editor Comments (if provided):


[Note: HTML markup is below. Please do not edit.]

Reviewers' comments:

Reviewer's Responses to Questions

**Comments to the Author**

1. Does this manuscript meet PLOS Global Public Health’s [publication criteria](https://c05y1x9s.r.us-east-2.awstrack.me/L0/https:%2F%2Fjournals.plos.org%2Fglobalpublichealth%2Fs%2Fcriteria-for-publication/1/010f0192268ee891-5f7de295-e828-4523-ba14-2a6b6ab5d7c0-000000/o80Da-yNQ9nTs5MmCb-vrEBIt5c=178)? Is the manuscript technically sound, and do the data support the conclusions? The manuscript must describe methodologically and ethically rigorous research with conclusions that are appropriately drawn based on the data presented.

Reviewer #1: Partly

2. Has the statistical analysis been performed appropriately and rigorously?

Reviewer #1: No

3. Have the authors made all data underlying the findings in their manuscript fully available (please refer to the Data Availability Statement at the start of the manuscript PDF file)?

The [PLOS Data policy](https://c05y1x9s.r.us-east-2.awstrack.me/L0/https:%2F%2Fjournals.plos.org%2Fglobalpublichealth%2Fs%2Fdata-availability/1/010f0192268ee891-5f7de295-e828-4523-ba14-2a6b6ab5d7c0-000000/L2_MSvF6GEjP9pWqVBq6rt3AkbA=178) requires authors to make all data underlying the findings described in their manuscript fully available without restriction, with rare exception. The data should be provided as part of the manuscript or its supporting information, or deposited to a public repository. For example, in addition to summary statistics, the data points behind means, medians and variance measures should be available. If there are restrictions on publicly sharing data—e.g. participant privacy or use of data from a third party—those must be specified.

Reviewer #1: No

**Response:**

We have added the supporting dataset as an attachment to this submission as a Supporting Information file.(S2 file)

4. Is the manuscript presented in an intelligible fashion and written in standard English?

PLOS Global Public Health does not copyedit accepted manuscripts, so the language in submitted articles must be clear, correct, and unambiguous. Any typographical or grammatical errors should be corrected at revision, so please note any specific errors here.

Reviewer #1: Yes

5. Review Comments to the Author

Please use the space provided to explain your answers to the questions above. You may also include additional comments for the author, including concerns about dual publication, research ethics, or publication ethics. (Please upload your review as an attachment if it exceeds 20,000 characters)

Reviewer #1: The manuscript analyses original data through a rather widely-used approach. Though the research questions are well posed, I cast some doubts about the employed methods. More details and sensitivity analyses are require in general. Detailed comments follow.

**Response**

We have reconducted the data analysis using cumulative ordinal mixed models and conducted sensitivity analysis. We conducted sensitivity analysis first by using ordinal multilevel ordinal Bayesian models enabling us to test for equal variance and proportion odds assumption and how they affect out model fit. We developed 4 models in the process.

Model1: Bayesian cumulative ordinal model with Proportional odds assumption

Model 2: Category-specific effects model using adjacent-category method

Model 3: Unequal variances model with unequal variance in selected variables

Model 3a: Unequal variances model with unequal variance in all covariates

Model 3b: Unequal variances model with unequal variance in all covariates +interaction term

Model 4: Adjacent-category model without category-specific effects.

We then conducted additional sensitivity analysis. we assessed how changes in random effect and cluster sizes affect our study outcomes. We first evaluated how our results varied when our model only included clusters as the only random effect, both cluster and individual participant as random effects and when no random effect was included. Secondly, we excluded study clusters with less than 5 people followed by exclusion of clusters with less than 10 participants and assessed the changes in our results. (Methodology Pages 12-16 and results pages 20-26).

1. The data and the code used to perform the analysis are not attached to the manuscript. Please, add both as supplementary material to ensure the reproducibility of the results.

**Response**

We have added the data and R codebooks associated with the analysis performed as supplementary files (S2 file).

2. Up to what I understand, the outcomes are measured on a discrete scale. If so, it is rather unclear to me why linear models are considered, as they are thought for continuous variables. I suggest to look at generalized linear (mixed) models instead; these would better reflect the proposed categorization described from line 290, where essentially categorical ordinal outcomes are defined.

**Response**

As recommended, we have used the multilevel cumulative ordinal mixed methods in the re-analysis of the data and conducted sensitivity analysis compared the cumulative model to category specific and unequal variance models (Page 13-16). In so doing, we have based more on the recommendations by Bürkner et al (2019).

Bürkner P-C, Vuorre M. Ordinal Regression Models in Psychology: A Tutorial. Advances in Methods and Practices in Psychological Science. 2019;2(1):77-101

3. Bearing in mind my previous comment, no matter which modelling or testing approaches are considered, the assumptions on which the employed methods are based should be carefully checked. For example, if a t-test is applied, please provide evidence that outliers are not present, homoscedasticity is met, gaussianity is a plausible assumption, etc; similarly, in a regression context the Gauss-Markov assumptions must be checked. In general, more details are required. Is the random effects distribution Gaussian? If so, how many quadrature points are considered? What's the estimate of the random effects’ variance? Is this assumption plausible? Why random slopes are not considered? Are you sure that all the fitted values are between the minimum and the maximum plausible values? Please, justify all the modelling choices.

**Response**

We have considered the cumulative ordinal mixed model assumptions and tested them. Especially for proportional odds assumption and equal variance assumption, we have compared models where these assumptions hold (model1 to those where these assumptions are relaxed or does not apply (model 2,3,4) and provide the results for each of these models in S1 file. We have provided the codes for the approaches we used in testing these assumptions. (S2 file)

4. Complete cases analysis is known to produce biased estimates. I am a bit puzzled on the your statement that missingness is completely non-at-random, how do you test it? I feel that methods such as pattern mixtures or selection models should be further considered and sensitivity analyses performed. This book could be a good reference <https://www.routledge.com/Handbook-of-Missing-Data-Methodology/Molenberghs-Fitzmaurice-Kenward-Tsiatis-Verbeke/p/book/9780367739294?srsltid=AfmBOormTtlUOXkGjISUZcE9WCOAlcFs60X0PSaQktOihmf_Mn_qWYxw>, but please be aware that the literature on this matter is massive.

**Response**

We have used intention-to-treat analysis instead. We have rechecked our data “missingness” and concluded it is Missing-at-random (MAR) as “missingness” was related to the observable study variables hence plausibility of MAR. We have provided the rationale in the methodology section. We have also provided the imputation codebook including exploratory analysis done to explore missingness (S2 file).

5. I see that multiple imputation is considered instead. More details are required on this as well. How datasets were considered? Did you check for the appropriateness of the imputations? Please, take <https://www.jstatsoft.org/article/view/v045i03> as reference.

**Response**

We have also provided the imputation codebook, including exploratory analysis done to explore missingness and analysis done to check appropriateness of the imputation (S2 file).

6. Please, provide the estimated coefficients for the confounders as well. Did you estimate any differences across cities?

**Response**

We have provided the estimates for all variables controlled for in the models in our edited tables. We have controlled for the town in our analysis. There were significant differences in the two towns. We attempted to do analysis per town but our sample size could not support considering our main outcome of interest involved interaction between time and allocation group. Hence, we decided to control for it instead in the analysis. (Table 5 and 6).

6. PLOS authors have the option to publish the peer review history of their article ([what does this mean?](https://c05y1x9s.r.us-east-2.awstrack.me/L0/https:%2F%2Fjournals.plos.org%2Fglobalpublichealth%2Fs%2Feditorial-and-peer-review-process%23loc-peer-review-history/1/010f0192268ee891-5f7de295-e828-4523-ba14-2a6b6ab5d7c0-000000/XgYPu7q75-qRpI06hjmFXU2uLSs=178)). If published, this will include your full peer review and any attached files.

**Do you want your identity to be public for this peer review?** If you choose “no”, your identity will remain anonymous but your review may still be made public.


For information about this choice, including consent withdrawal, please see our [Privacy Policy](https://c05y1x9s.r.us-east-2.awstrack.me/L0/https:%2F%2Fwww.plos.org%2Fprivacy-policy/1/010f0192268ee891-5f7de295-e828-4523-ba14-2a6b6ab5d7c0-000000/xGk7uBH4DmS0mSd5K04-NrRWTBY=178).

Reviewer #1: No

[NOTE: If reviewer comments were submitted as an attachment file, they will be attached to this email and accessible via the submission site. Please log into your account, locate the manuscript record, and check for the action link "View Attachments". If this link does not appear, there are no attachment files.]

While revising your submission, please upload your figure files to the Preflight Analysis and Conversion Engine (PACE) digital diagnostic tool, <https://pacev2.apexcovantage.com/>. PACE helps ensure that figures meet PLOS requirements. To use PACE, you must first register as a user. Registration is free. Then, login and navigate to the UPLOAD tab, where you will find detailed instructions on how to use the tool. If you encounter any issues or have any questions when using PACE, please email PLOS at [figures@plos.org](mailto:figures@plos.org). Please note that Supporting Information files do not need this step.
