# Supplementary material for: Effectiveness of barber-facilitated “Doing What Matters in Times of Stress” intervention among urban literate youths in Western Kenya: A cluster randomised trial
Source: PLOS Glob Public Health. 2025 Jun 18;5(6):e0004712. doi: 10.1371/journal.pgph.0004712 (PMC12176197; doi:10.1371/journal.pgph.0004712)
Supplement: Attachment — Submitted filename: Response to reviewers comments11_4_25.docx [file pgph.0004712.s017.docx]

11^th^ April 2025.

Dr Joel Msafiri Francis, PhD

Academic Editor

PLOS Global Public Health

Dear Dr Joel Msafiri,

**Ref: Address to reviewers’ comments**

I hope this message finds you well. I would like to express our gratitude for the opportunity to revise our manuscript titled “Effectiveness of Barber-Facilitated "Doing What Matters in Times of Stress" Intervention Among Urban Literate Youths in Western Kenya: A cluster randomised trial” (PGPH-D-24-01544R1) following the insightful comments from the reviewer.

We appreciate the time and effort the reviewer took to provide detailed feedback on our revised manuscript. Their comments were invaluable in strengthening the manuscript. Below, we have addressed each of the reviewers' points and where necessary made changes in track changes in the manuscript:

**Journal Requirements:**

Please review your reference list to ensure that it is complete and correct. If you have cited papers that have been retracted, please include the rationale for doing so in the manuscript text, or remove these references and replace them with relevant current references. Any changes to the reference list should be mentioned in the rebuttal letter that accompanies your revised manuscript. If you need to cite a retracted article, indicate the article’s retracted status in the References list and also include a citation and full reference for the retraction notice.

**We have reviewed all included references. We have checked each of the references from the journal websites and none has been retracted.**

**We have edited one reference on GAD-7 scoring, replacing the review article we had cited to the original article by Spitzer et al., 2006. (line 281).**

**We have also edited reference for line 73, replacing the reference with the most appropriate one on the original report on common mental health disorders in Kenya (***Ministry of Health. Mental health task force report - mental health and wellbeing towards happiness & national prosperity - the taskforce on mental health. 2020. Nairobi, Kenya: Ministry of Health; 2020)* **(line 73).**

**Comments to the Author**


2) Discussion & Conclusion:
The author concludes that “barbershops are potentially safe and feasible community places to promote mental health awareness and prevent mental disorders...” While DWM intervention appears to be low-intensity and potentially safe, concluding definitively on its safety based solely on the findings from this study warrants caution.
There is literature, including anecdotal and research-based reports (see, for example: Chen X, Stanton B, Li X, Fang X, Lin D. Substance use among rural-to-urban migrants in China: a moderation effect model analysis. Subst Use Misuse. 2008;43(1):105-24. doi: 10.1080/10826080701209077. PMID: 18189208 and Barlow, A., & Hawdon, J. (2015). Sex, Drugs, and Deception: Deviance in the Hair Salon Industry. Deviant Behavior, 37(1), 66–80. https://doi.org/10.1080/01639625.2014.983009), suggesting that barbershops and hair salons may, in some instances, also serve as hotspots sites for activities such as substance use and sex work. Given this context, a more nuanced discussion around the safety of barbershops as intervention spaces is warranted.

To strengthen the conclusion, it would be helpful for the author to indicate whether any aspects of safety—including the potential for such adverse contextual factors—were observed or noted in the study or referenced from the literature. Furthermore, ascertain whether there were any serious adverse events (SAE) reported in the course of the study. Insights in this regard would enhance the credibility of the safety claims, particularly considering that some follow-up visits occurred at locations chosen by the participants in agreement with the barber.
If no data on adverse events is available, this should be acknowledged as a limitation, given its implications for future scale-up. Additionally, any safeguards integrated into the DWM barber training or study monitoring process should be highlighted to reassure readers and stakeholders of the ethical and safety frameworks underpinning the intervention.

**We monitored data on any adverse events in our process evaluation of the project and none such events was reported or identified with regards to use of barbers and barbershops. We have highlighted the safeguard measures put in place to ensure safety of the barbers and participants in supplementary text S2.**

*(The barbers delivered the intervention with daily supervision by the project psychologist for the first week to ensure they did it correctly. After that, we had weekly support supervision by the project psychologist. Supervision ensured the quality delivery of the intended intervention and prevented helpers’ burnout and further, assisted barbers in feeling supported and confident with dealing with the clients***.)**

**Besides, in the conclusion we emphasise that Its implementation requires adequate capacity building and supervision by trained mental health specialists as a safeguard against any unforeseen adverse effects. Despite there being no such adverse events reported from our intervention monitoring, we agree that there is likelihood of such adverse events occurring and highlight the need for safeguards.**

***Its implementation requires adequate capacity building and supervision by trained mental health specialists to address any potential adverse events and an integrated stepped-care framework where those with severe mental health disorders are referred to appropriate clinical interventions.*** Line 601 -604

3) Statistical Analysis:
The author has addressed several concerns from the initial review. However, with respect to the question about the Missing at Random (MAR) assumption, the opening statement in the response—“We have used intention-to-treat analysis instead”—is misapplied. Intention-to-treat (ITT) is a study design principle that ensures participants are analyzed in the groups to which they were randomized; it is not a statistical method that addresses the missing data mechanism, nor does it justify assumptions regarding MAR.
If the assumption is that data were MAR due to their association with observed covariates, the authors should specify which covariates were predictive of missingness and how this informed the MAR assumption. Clarifying this point would strengthen the robustness of the statistical handling of missing data and provide greater transparency in the analysis.

**We have revised this. We provide detailed information of the tests we did for missingness assumption. We provide details of analysis done to test plausibility of MAR assumption and provide examples of variables that were significantly associated with missingness for our outcomes for us to believe that MAR was plausible. We also provide the analysis codebook which contains the code for the test carried out for missingness assumption. (and supplementary files S2 file).**

**We have also edited the analysis section to provide the below details of the missingness test done to conclude that data was MAR.**

***(We visualised the data for missingness patterns. We tested if the data was missing completely at random (MCAR) using the TestMCARNormality function from the MissMech package (41). We used missing_compare() function from the finalfit package (42) to test if missingness for each of our outcome variable was associated with any of the explanatory variables in our study. Several of our explanatory variables significantly predicted missingness in our outcomes, suggesting plausibility of MAR assumption. For example, missingness in depression scores was significantly associated with allocation group, time point of assessment and town of residence.***

***We also used logistics regression to model the probability of missingness for each outcome as a function of the explanatory variables in the study. For each outcome, we created a binary variable missing outcome, “true” if outcome score was missing, “false” otherwise. We used this outcome for the logistics regression. A number of the explanatory variables were significant predictors of missingness. For example, for all outcomes, allocated group and town of residence were significant predictors of missingness, supporting plausibility of MAR assumption. (Lines 373-387)***

While the manuscript presents the findings in a reasonably understandable manner, the author should consider expanding the explanation and evidence supporting their analytical choices in the revised version.

**We have provided detailed justification and evidence supporting our analytical choice, the decision to use ordinal models and not linear mixed effect models. (lines 294-310)**

***(We first fitted*** ***multilevel linear mixed effect model to our outcome scores. However, our outcomes and resulting model outputs did not meet the model assumptions of linear relationship between predictors and outcome and normality of residuals. Besides, our outcome scores were not continuous variables in nature. Hence, we did not consider this model appropriate.***

***We used ordinal models instead, since ordinal outcome assessment tools were administered. The resulting scores from such tools have been shown to routinely violate the normality distributional assumption of linear models (37). The use of linear models treats ordinal data as equal interval values, when in practice, differences from one ordinal category to another vary (37, 38). For example, for PHQ-9 tool, responses can vary as Not at all=0, Several days=1, More than half the days=2 and Nearly every day=3, in an ordinal way, though the psychological distances between them are not guaranteed as equal. Treating scores from such scales as if normally-distributed equal-interval continuous values is considered inappropriate (37). Such practices can result in systemic errors, inflate type I error and distort estimate of effect size (37). Use of linear or nominal models for such data leads to distortion of the result, hence it is recommended to use ordinal models for such datasets instead (37, 38). This informed our choice for ordinal models for our data analysis). Lines 294-310***

4) Editorial Comments:
Lines 237–241 should be moved to follow the paragraph describing the “waiting list arm procedure” (currently ending at line 248). Logically, it would be clearer to describe the control group procedures before discussing data collection and follow-up related to that group.

**We have moved the former line 237-241 to after the description of the waiting list arm. We have given it a subheading Outcome assessment and follow up. (line 244-251)**

[NOTE: If reviewer comments were submitted as an attachment file, they will be attached to this email and accessible via the submission site. Please log into your account, locate the manuscript record, and check for the action link "View Attachments". If this link does not appear, there are no attachment files.]

While revising your submission, please upload your figure files to the Preflight Analysis and Conversion Engine (PACE) digital diagnostic tool, https://pacev2.apexcovantage.com/. PACE helps ensure that figures meet PLOS requirements. To use PACE, you must first register as a user. Registration is free. Then, login and navigate to the UPLOAD tab, where you will find detailed instructions on how to use the tool. If you encounter any issues or have any questions when using PACE, please email PLOS at figures@plos.org. Please note that Supporting Information files do not need this step

**We uploaded all our figure files in PACE and ensured that they are acceptable as per the pace guidelines.**

Additional edits.

**All authors have proof read and grammatically edited and formatted the whole manuscript.**

Protus Musotsi Yabunga

For all authors.
